# 3D Bioprinted Artificial Trachea with Epithelial Cells and Chondrogenic-Differentiated Bone Marrow-Derived Mesenchymal Stem Cells

**DOI:** 10.3390/ijms19061624

**Published:** 2018-05-31

**Authors:** Sang-Woo Bae, Kang-Woog Lee, Jae-Hyun Park, JunHee Lee, Cho-Rok Jung, JunJie Yu, Hwi-Yool Kim, Dae-Hyun Kim

**Affiliations:** 1 Department of Veterinary Surgery, College of Veterinary Medicine, Konkuk University, 120 Neungdong-ro, Gwangjin-gu, Seoul 05029, Korea; raphael0826@gmail.com (S.-W.B.); planetes1202@naver.com (J.-H.P.); hykim@konkuk.ac.kr (H.-Y.K.); 2 Division of Cardiovascular Surgery, Severance Cardiovascular Hospital, Yonsei University College of Medicine, 50-1 Yonsei-ro, Seodaemun-gu, Seoul 03722, Korea; sysebg@naver.com; 3 Department of Nature-Inspired Nanoconvergence System, Korea Institute of Machinery and Materials (KIMM), 156 Gajeongbuk-Ro, Yuseong-Gu, Daejeon 34103, Korea; meek@kimm.re.kr (J.L.); junjie0801@kimm.re.kr (J.Y.); 4 Gene Therapy Research Unit, Korea Research Institute of Bioscience and Biotechnology, 125 Gwahak-ro, Yuseong-gu, Daejeon 34141, Korea; crjung@kribb.re.kr; 5 Department of Biomedical Engineering, School of Integrative Engineering, Chung-Ang University, 84 Heukseok-Ro, Dongjak-Gu, Seoul 06974, Korea

**Keywords:** bone marrow-derived mesenchymal stem cell, chondrogenic differentiation, three-dimensional bioprinting, artificial trachea, tissue engineering

## Abstract

Tracheal resection has limited applicability. Although various tracheal replacement strategies were performed using artificial prosthesis, synthetic stents and tissue transplantation, the best method in tracheal reconstruction remains to be identified. Recent advances in tissue engineering enabled 3D bioprinting using various biocompatible materials including living cells, thereby making the product clinically applicable. Moreover, clinical interest in mesenchymal stem cell has dramatically increased. Here, rabbit bone marrow-derived mesenchymal stem cells (bMSC) and rabbit respiratory epithelial cells were cultured. The chondrogenic differentiation level of bMSC cultured in regular media (MSC) and that in chondrogenic media (d-MSC) were compared. Dual cell-containing artificial trachea were manufactured using a 3D bioprinting method with epithelial cells and undifferentiated bMSC (MSC group, *n* = 6) or with epithelial cells and chondrogenic-differentiated bMSC (d-MSC group, *n* = 6). d-MSC showed a relatively higher level of glycosaminoglycan (GAG) accumulation and chondrogenic marker gene expression than MSC in vitro. Neo-epithelialization and neo-vascularization were observed in all groups in vivo but neo-cartilage formation was only noted in d-MSC. The epithelial cells in the 3D bioprinted artificial trachea were effective in respiratory epithelium regeneration. Chondrogenic-differentiated bMSC had more neo-cartilage formation potential in a short period. Nevertheless, the cartilage formation was observed only in a localized area.

## 1. Introduction

The trachea is a hollow cylindrical organ composed of 15–20 C-shaped cartilages with fibroelastic ligaments [1]. Permanent damage, stenosis and tumor in the trachea require surgical intervention with tracheal resection [2] and in such cases, circumferential resection and end-to-end anastomosis are generally considered the optimal surgical methods. However, these procedures are applicable only in particular conditions. For instance, when a lesion exceeds half of the trachea in adults, or one third in children, tracheal replacement is recommended rather than tracheal resection. Nevertheless, with the developments in tissue engineering, tracheal replacement strategies were diversified from the use of prostheses and synthetic stents to tissue transplantation [3].

The first case of allogenic tracheal transplantation in humans was published by Rose et al. in 1979 [4]. A cadaveric tracheal graft was heterotopically transplanted for 3 weeks to provide revascularization and subsequently orthotopically repositioned. Wurtz et al. reported on tracheal replacement with an aortic homograft combined with an intraluminal stent to support the structural integrity [5]. Macroscopically, the aortic graft was surrounded by a thick wall including recognizable cartilage rings; however, progressive ischemia of the cartilage was observed pathologically. Moreover, artificial prostheses have also been used for tracheal replacement; however, they have been associated with material migration, rupture, infection and disintegration [6]. Hence, no satisfying methods in the aspect of multilayered structure and its function in tracheal reconstruction have been identified [7,8,9]. 

Three-dimensional (3D) bioprinting serves as an additional manufacturing method that uses a 3D bioprinter and enables three-dimensional stacking of certain biomaterials layer by layer according to the intended design. Recent advances in tissue engineering enabled 3D bioprinting using various biocompatible materials including living cells, thereby making the product clinically applicable [10]. Additionally, mesenchymal stem cells (MSC), originally isolated from the bone marrow, are a promising cell type for tissue engineering applications because of their proliferation ability and their potential to differentiate in vitro and in vivo into multiple functional tissue-specific cell types, such as adipocytes, chondrocytes, osteoblasts and skeletal myocytes [11,12].

In this study, we compared the glycosaminoglycan (GAG) synthesis and chondrogenic differentiation level between two different culture methods: bMSC cultured in general medium (MSC group) and chondrogenic-differentiated bMSC cultured in chondrogenic medium (d-MSC group). Using a 3D bioprinter, we developed a novel biocompatible artificial trachea with epithelial cells + bMSC (MSC group) and with epithelial cells + chondrogenic-differentiated bMSC (d-MSC group). The structure of the artificial trachea was considered to be similar to that of the normal trachea. We evaluated the results obtained from the animal study according to the chondrogenic differentiation level of bMSC.

## 2. Results

### 2.1. Relative Quantification of Glycosaminoglycan (GAG) in bMSC and Chondrogenic-Differentiated bMSC

**Figure 1:**
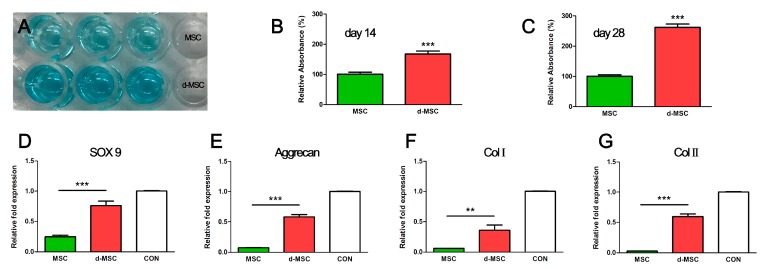
Relative GAG accumulation and chondrogenic marker gene expression level of MSC and d-MSC. (**A**) Alcian blue-stained dissolvents in a 96-well assay plate; the d-MSC group showed a deeper blue color than the MSC group; (**B**,**C**) Alcian blue absorbance on days 14 and 28. The d-MSC group showed 1.67 ± 0.10 (day 14) and 2.62 ± 0.11 (day 28) times higher values than the MSC group. The relative gene expression levels of SOX9 (**D**); aggrecan (**E**); collagen type 1 (**F**) and collagen type 2 (**G**) were increased in d-MSC. CON = expression level of chondrocyte as positive control. ** *p* < 0.01, *** *p* < 0.001.

Relative quantification of GAG between bMSC and chondrogenic-differentiated bMSC on days 14 and 28 was compared. The relative values of chondrogenic-differentiated bMSC on days 14 and 28 were, respectively, 1.67 ± 0.10 and 2.62 ± 0.11 times higher than those of bMSC (Figure 1). 

### 2.2. Chondrogenic Gene Expression

Gene expression levels were normalized to glyceraldehyde-3-phosphate dehydrogenase (GAPDH) and calculated as the expression relative to that of rabbit ear cartilage cell as control. Quantitative real-time PCR (qRT-PCR) analysis demonstrated that the expression levels of all chondrogenic genes (*ACAN*, *Col1α1*, Col2α1 and *SOX9*) in d-MSC were higher than those in MSC. The expression level of Col2α1 significantly increased 22.54 fold ± 4.24 in d-MSC. *Aggrecan* (*ACAN*), Col1α1 and SOX9 gene expression levels were also increased (7.71 ± 0.75, 5.93 ± 1.38 and 3.10 fold ± 0.41, respectively) in d-MSC compared with those in MSC (Figure 1).

### 2.3. Structure of the 3D Bioprinted Artificial Trachea

**Figure 2:**
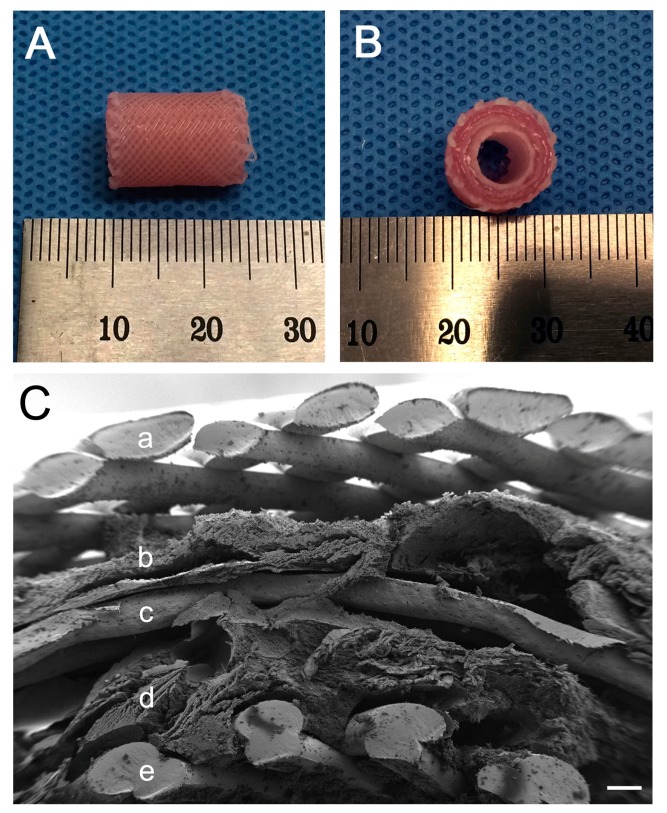
3D bioprinted artificial trachea. (**A**) Longitudinal view, 15 mm in length; (**B**) Vertical view, five-layered structure with a 5-mm inner diameter and 10-mm outer diameter; (**C**) Magnified structure of the scaffold (SEM image). (a,c,e) layers are composed of PCL. (b) is alginate layer with MSC or d-MSC and (d) is alginate layer with epithelial cells. The innermost (e) and outermost (a) layers showed a microporous feature. The non-porous third layer (c) separates the epithelial cell layer (d) and the MSC or d-MSC layer (b). The scale bar indicates 200 µm.

**Figure 3:**
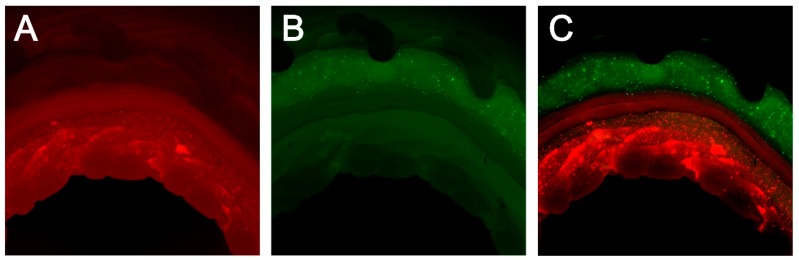
Cell distribution in alginate hydrogel with CellTracker™. (**A**) Epithelial cell in the second alginate hydrogel layer (red); (**B**) MSC in fourth alginate hydrogel layer (green); (**C**) A and B (merged). Each cell was well-printed layer by layer and separated completely.

The 3D bioprinted artificial trachea was well manufactured (Figure 2). The SEM image revealed micropores in the innermost and outermost PCL layers and showed that the middle non-pore PCL layer separates the two alginate hydrogel layers (Figure 2). Observation of the artificial trachea stained with the CellTracker™ (Thermo Fisher Scientific, Waltham, MA, USA) under a light microscope with optical filters showed a complete separation of inner layer epithelial cells (stained red) and outer layer bMSC (stained green) (Figure 3). All cells were evenly scattered in the alginate hydrogel.

### 2.4. Application of the 3D Bioprinted Artificial Trachea

**Figure 4:**
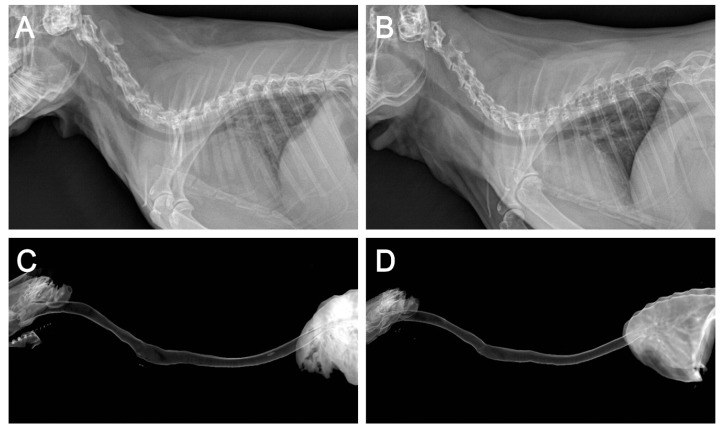
Radiographic findings. Plain lateral thoracic view of the MSC (**A**) and d-MSC (**B**) groups and 3D reconstructed CT image of the MSC (**C**) and d-MSC (**D**) groups at 12 weeks after 3D bioprinted artificial trachea transplantation. Well-sustained tracheal contour was observed with no signs of stenosis or obstruction.

**Figure 5:**
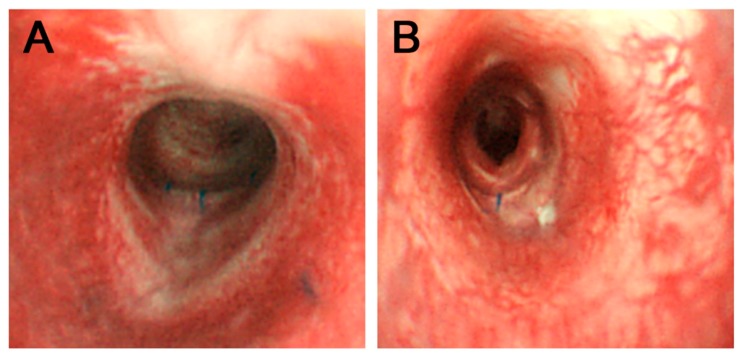
Bronchoscopic findings. Bronchoscopic images of MSC group (**A**) and d-MSC group (**B**) were obtained 12 weeks after the surgery. Tracheal lumen fully covered with epithelial mucosa was observed. Remaining suture materials indicate the implant fixation site.

All rabbits that underwent surgery showed no signs of respiratory distress, graft failure, or infection until sacrifice at 12 weeks postoperatively. Plain thoracic radiographs of the MSC and d-MSC groups demonstrated properly sustained tracheal contour without obstruction or stenosis and the 3D-reconstructed CT images indicated good luminal contour of the trachea and no signs of stenosis (Figure 4). The bronchoscopic examination revealed that the tracheal defect was fully re-covered with epithelial mucosa during the experimental period (Figure 5). 

### 2.5. In Vivo Epithelialization and Neo-Cartilage Formation

**Figure 6:**
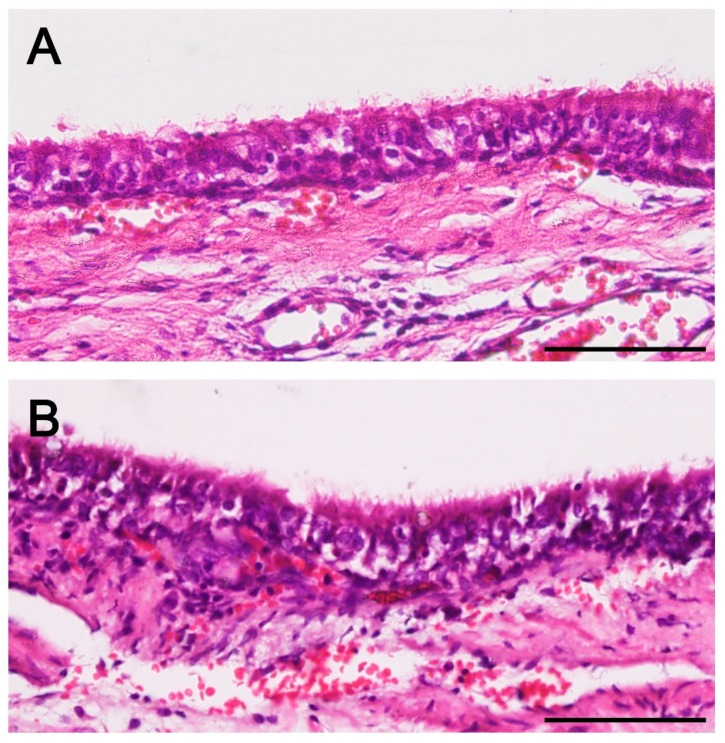

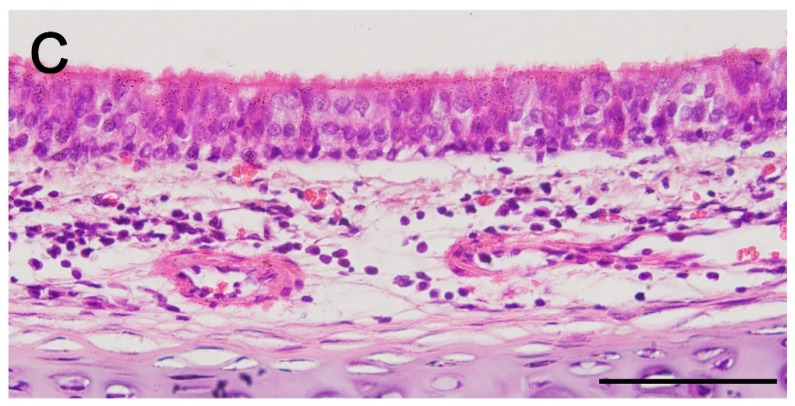
Histopathologic findings. Microscopic images of MSC group (**A**) and d-MSC group (**B**) showed newly formed respiratory epithelium in both groups. Regenerated epithelium in A and B had a rough cell arrangement compared with that of the normal trachea (**C**) but ciliated columnar epithelium was confirmed (Hematoxylin and eosin staining; all scale bars indicate 50 µm).

**Figure 7:**
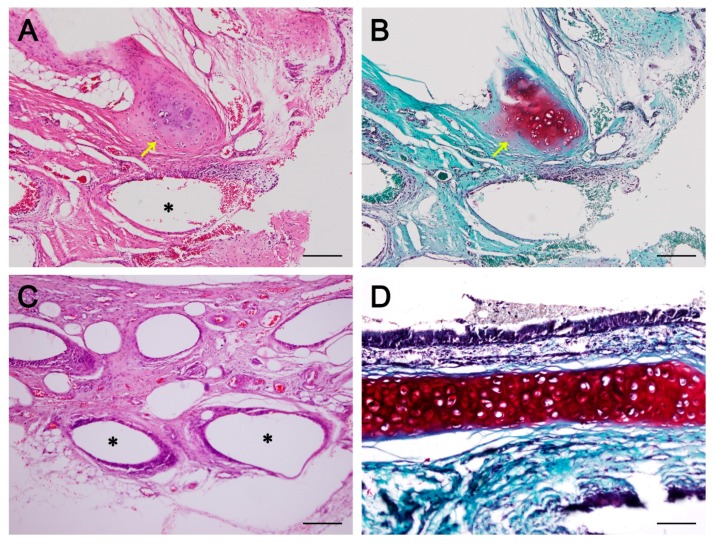
Neo-cartilage formation and neo-vascularization. Neo-vascularization and neo-cartilage formation was observed in the d-MSC group (**A**,**B**). However, in the MSC group, neo-vascularization was seen but no cartilaginous islet was observed (**C**). Newly formed immature cartilage islet (yellow arrows) had higher cellular density and lighter proteoglycan staining compared with the cartilage of the normal trachea (**D**). A, C and D = hematoxylin and eosin staining; B = safranin O staining. Asterisks (*) indicate fifth polycaprolactone (PCL) layer and all scale bars indicates 50 µm).

On histopathologic analysis, newly formed respiratory epithelium at the implant regions was observed in all groups. Hematoxylin and eosin (H&E) staining showed that the newly formed epithelium appeared a little rough compared with the normal respiratory epithelium; nevertheless, it has a quite organized structure with cilia (Figure 6). Abundant neovascularization was also found around the implants in all groups. However, neo-cartilage formation was limited to d-MSC group only. On safranin-O/fast green staining, the newly formed cartilage had higher density in cellularity and lighter proteoglycan staining than the normal mature cartilage. The newly formed cartilage was not lining the whole contour of the trachea and was thus limited to a localized area (Figure 7).

## 3. Discussion

In this study, we cultured rabbit bMSC in a chondrogenic medium and their chondrocyte-like characteristics were identified by modified alcian blue absorbance test and qRT-PCR. Epithelialization and neo-cartilage formation were observed in the animal transplant experiment using a 3D bioprinted artificial trachea, which has autologous epithelial cells and chondrogenic-differentiated bMSC.

Aggrecan, Col2α1 and *SOX-9* are known as chondrogenic differentiation markers; *SOX-*9 is pre-chondrogenic marker [13]. Lefebvre et al. identified that *SOX-9* activate aggrecan and Col2α1 genes in cultured cells; hence, it plays an essential role in chondrogenic differentiation [14]. In our study, *SOX-9*, aggrecan, Col1α1 and Col2α1 gene expressions were upregulated in the d-MSC group compared with those in the MSC group. This result is similar to that reported in Bo Wei et al.’s in vitro study of bone marrow MSC-derived extracellular matrix. They revealed that a greater increase in cartilage-like gene expression was observed in the group cultured with transforming growth factor (TGF)-β [15]. Moreover, the qRT-PCR gene expression results indicate that d-bMSC have a greater possibility to give rise to chondrogenic tissue formation than bMSC, which is supported by Barry et al. who demonstrated that TGF could induce chondrogenic differentiation in mesenchymal stem cells [16]. Kojima et al. also demonstrated neo-cartilage formation with supplemental TGF-β in bMSC polymer tissue-engineered trachea and its GAG content levels were also similar to those of a normal cartilage [17]. In our study, the relative accumulation of GAG, assessed by modified alcian blue staining, in cultured cells also supports the qRT-PCR result that chondrogenic-differentiated bMSC have more potency in synthesizing GAG contents than bMSC. 

Various materials, that is, from synthetic products to biological tissues, were used for tracheal tissue reconstruction; however, previous reports with sufficiently successful outcomes are limited. Behrend et al. reported in a study of homogenic tracheal transplantation in sheep that the grafts were completely absorbed and replaced by inflammatory scar tissue; thus, the stability of the trachea could not be secured [18]. A study of tissue-engineered allograft, using fibrin-hyaluronan composite gel and chondrocyte, by Kim et al. showed partial success, with fine luminal contour of the regenerated site but insufficient neo-cartilage formation [19]. For a successful tracheal tissue transplantation, the following requirements for the grafts are inevitable: appropriate mechanical properties, adequate blood supply to maintain the characteristic structure and lining with ciliated epithelium [6,7,20]. Several studies showed that bMSC promotes neo-angiogenesis. Han et al. demonstrated that bMSC may enhance neo-vascularization in cryopreserved trachea allograft by upregulating vascular endothelial growth factor expression [21]. Hence, in our study, the abundant neo-vascularization was associated with the angiogenic potential of bMSC.

Patrício et al. demonstrated that PCL/PLA (poly lactic acid) scaffolds produced by solvent casting showed a better result in reduced pore size, mechanical properties and cell adhesion than PCL/PLA scaffolds produced by melt blending [22]. In another recent study, PCL/HA (hydroxyapatite) scaffolds produced using an extrusion-based system revealed successful composite scaffold with fully interconnected pores [23]. Biodegradable materials such as polyglycolic acid (PGA), polylactic acid (PLA), poly(lactic-co-glycolic) acid (PLGA) and polycaprolactone (PCL) used for 3D printing have strength corresponding to the tracheal cartilage and therefore various attempts are being made to apply 3D bioprinting technology to tracheal transplant research [1]. Moreover, recent advances in 3D bioprinting techniques resulted in hybridization of scaffolds with various cells, including mesenchymal stem cells. Zopf et al. reported the use of a customized 3D-printed, biodegradable tracheal prosthesis made with PCL in a patient with tracheobronchomalacia [24]. PCL is a synthetic bioresorbable polymer with potential applications in tissue engineering and thus can be used in a 3D bioprinter without deleterious solvents. It also has excellent mechanical properties and slow degradation in vivo via enzymatic hydrolysis [9]. Moreover, Son et al. demonstrated that a PCL/poly(methyl methacrylate) scaffold is appropriate for neo-bone formation in vivo and cell growth in vitro [25]. Costantini et al. showed that 3D bioprinted scaffolds with bone marrow-derived human mesenchymal stem cells in alginate, as a templating agent for stability during 3D printing, exhibit enhanced chondrogenic differentiation of bMSC in a chondrogenic medium. Additionally, mixing cells with alginate enabled the formulation of biomimetic inks for 3D printing, which in turn could be used in cartilage tissue engineering [26].

In an animal experiment by Go et al., decellularized matrix tracheal tissue transplantation was performed in pigs [27]. They reported that the experimental group, which was transplanted with autologous cells (inner autologous epithelial cells and outer autologous bMSC-derived chondrocytes), had a significantly higher survival rate with no signs of airway collapse or ischemia than the other groups. Furthermore, biological tissues are composed of more than two types of cells. Hence, if scaffolds contain two or more cells, they become more similar to biological tissues and could be more effective in vivo. Currently, having multiple cells in one scaffold is challenging and several investigators have been attempting to overcome this limitation. The advancement in 3D bioprinting techniques with hydrogels is considered an extremely effective solution for various kinds of cells to be applied to a scaffold [28].

As previously mentioned, the trachea is a hollow cylindrical organ with outer C-shaped cartilages covered with respiratory epithelium inside. For tracheal reconstruction, the scaffold should be a favorable environment for respiratory epithelium and neo-angiogenesis and its shape and strength must be appropriate [1]. The artificial trachea used in this study was cylindrical in shape, which is similar to the normal trachea, with five multilayers composed of biocompatible materials (i.e., PCL and alginate hydrogel). The three major aspects in tissue regeneration using tissue engineering include the scaffold, injection of cells and cells seeded or within the scaffold [29] and the last item was involved in our study. We believe that the artificial trachea we manufactured is novel; it contains two different types of cell in the alginate hydrogel separated by middle non-porous PCL layer for mechanical strength and has micropores in the innermost and outermost PCL layer for a smooth communication between cells and adjacent tissues. Furthermore, in our study, we observed newly formed respiratory epithelium in all groups; however, neo-cartilage formation was detected only in the d-MSC group and was limited in a localized area. During tracheal regeneration, cartilage formation is vital as well as epithelialization to maintain mechanical strength and function of the airway. Based on our experiment, chondrogenic differentiation of bMSC was more effective for tracheal cartilage regeneration than non-differentiated bMSC. Nonetheless, further study with a long-term observation and different cell application appears necessary in the near future. Moreover, mechanical test of the tracheal scaffold and dynamic air flow analysis throughout the respiratory system should be conducted prior to clinical application of tissue engineered tracheal replacement.

## 4. Materials and Methods

This animal study was approved by the Institutional Animal Care and Use Committee of Yonsei University Health System (publication no. 2015-0361, 2015). This study was performed according to the ARRIVE guidelines and the National Institutes of Health Guide for the Care and Use of Laboratory Animals. Approval date: 4 December 2015.

### 4.1. Primary Cell Culture

#### 4.1.1. Isolation and Culture of Autologous bMSC

Bone marrow-derived mesenchymal stem cells (bMSC) were isolated from each rabbit (New Zealand White rabbits, male, 3 months old; *n* = 6). Briefly, premedication with 5 mg/kg xylazine and 10 mg/kg Zoletil^®^ (Virbac Korea, Seoul, Korea) were administered intramuscularly and anesthesia was maintained by isoflurane inhalation. Bone marrow was harvested from the femur using a 13G bone biopsy needle and stored in a 50-mL pre-heparinized conical tube (SPL Life Sciences, Gyeonggi-do, Korea). The bone marrow was filtered through a 40-µm cell strainer (Life Sciences, New York, NY, USA) and mixed with phosphate-buffered saline (PBS) of up to 8 mL. The mixture was centrifuged at 1500 rpm for 5 min, the supernatant was discarded and the remaining precipitate was suspended with 8 mL serum-free Dulbecco’s Modified Eagle’s Medium (low glucose) (Welgene, Daegu, Korea). Subsequently, the mixture was transferred to a 15-mL conical tube (SPL Life Sciences) containing 6 mL of Ficoll-Paque^®^ (Sigma-Aldrich, St. Louis, MO, USA) and centrifuged at 1840 rpm for 30 min. After centrifugation, the interphase was harvested and mixed to the medium (up to 10 mL) in a 15-mL conical tube and centrifuged at 1500 rpm for 5 min and the supernatant was removed. The cell pellet was mixed with the medium supplemented with 10% fetal bovine serum (FBS) (GE Healthcare Life Sciences, Pittsburgh, PA, USA) and 1% penicillin-streptomycin (Thermo Fisher Scientific, Waltham, MA, USA). The culture medium was carefully changed after 3 days and every 2 days thereafter. The culture was maintained at 37 °C in a 5% CO_2_ incubator. The bMSC were passaged twice before the experiments. 

#### 4.1.2. Isolation and Chondrogenic Differentiation of Autologous bMSC (d-MSC)

The isolation procedure of bMSC was the same as that described above (New Zealand White rabbits, male, 3 months old; *n* = 6). For chondrogenic differentiation, 100 nM dexamethasone, 10% insulin-transferrin-selenium (ITS-premix), 1 µg/mL ascorbic acid, 1% sodium pyruvate, 10 ng/mL human transforming growth factor-β1 were added in the medium [12]. The culture medium was carefully changed after 3 days and every 2 days thereafter. The culture was maintained at 37 °C in a 5% CO_2_ incubator. The bMSC were passaged twice before the experiments.

#### 4.1.3. Isolation and Culture of Autologous Epithelial Cells

Epithelial cells were isolated from the rabbits described previously (New Zealand White rabbits, male, 3 months old; *n* = 12). A 4-mm skin biopsy punch was performed under general anesthesia and medial nasal mucosa was harvested from the nostril and stored in PBS containing 1% penicillin-streptomycin for 30 min. Subsequently, submucosal tissue was manually eliminated as much as possible on a sterilized petri dish. Remaining tissue explant was harvested, incubated with 0.2% (*w*/*v*) collagenase type II (Thermo Fisher Scientific) in Ham’s F-12 medium for 24 h, filtered through a 100-µm nylon cell strainer (BD Biosciences, Franklin Lakes, NJ, USA) and centrifuged at 1500 rpm for 5 min. After the supernatant was discarded, the cells were seeded on a 100-mm cell culture dish (SPL Life Sciences) with Ham’s F-12 medium (Welgene) supplemented with 10% FBS (GE Healthcare, Salt Lake City, UT, USA), 1% penicillin-streptomycin (Thermo Fisher Scientific), 10 µg/mL amphotericin B (Enzo Life Sciences, Farmingdale, NY, USA), 50 µg/mL gentamicin (Daesung Microbiological Labs, Gyeonggi-do, Korea), 0.5 µg/mL hydrocortisol (Sigma-Aldrich), 5 ng/mL epidermal growth factor (ProSpec, East Brunswick, NJ, USA), 1.5 µg/mL bovine serum albumin (MP Biomedicals, Santa Ana, CA, USA) and 1×ITS+3 solution (Sigma-Aldrich). Non-adherent cells were removed by washing in PBS and the culture medium was changed every 3 days. The culture was maintained at 37 °C in a 5% CO_2_ incubator. The epithelial cells were passaged twice before the experiments.

### 4.2. In Vitro Study

#### 4.2.1. Modified Alcian Blue Absorbance Test 

The relative quantity of GAG contents was determined using modified alcian blue absorbance test. bMSC and chondrogenic-differentiated bMSC were prepared at 5 × 10^4^ cells/mL in a 6-well cell culture plate. Non-adherent cells were removed by washing in PBS and the culture medium was changed every 3 days. The plates were maintained at 37 °C in a 5% CO_2_ incubator. On days 14 and 28, each well was washed with PBS twice and 20 µL of alcian blue solution was added into each well. After 15 min, the solutions were discarded and each well was washed with distilled water (DW) thrice. Subsequently, 350 µL of dimethyl sulfoxide (DMSO) was added into each well and the plates were placed on a cell culture rocker system for 10 min. Thereafter, 100 µL of the solution in each well was transferred to a 96-well assay plate (SPL Life Sciences). The absorbance at 610 nm was measured using a VersaMax ELISA Microplate Reader (Molecular Devices, San Jose, CA, USA) [13]. 

#### 4.2.2. RNA Extraction and Quantitative Reverse Transcription Polymerase Chain Reaction (qRT-PCR)

After 14 days of culture, total RNA of MSC and d-MSC was extracted using the MiniBest™ Universal RNA Extraction kit (TaKaRa Biomedicals, Otsu, Japan) according to the manufacturer’s protocol. The quantity and purity of the RNA from each sample were determined by the ratio of the optical density at 260 nm to that at 280 nm using Nanodrop™ (Thermo Fisher Scientific). Using PrimeScript™ RT reagent kit (TaKaRa Biomedicals), we prepared 1 µL of cDNA according to the manufacturer’s protocol. The PCR primers for aggrecan (ACAN), collagen type 1 (Col1α1), collagen type 2 (Col2α1) and SOX-9 (SOX9) gene are described in Table 1. qRT-PCR was performed to detect quantitative real-time PCR products from cDNA using SYBR Premix Ex Taq™ (TaKaRa Biomedicals). The qRT-PCR conditions were as follows: 35 cycles of denaturation at 95 °C for 30 s, annealing at 60 °C for 1 min and extension at 72 °C for 1 min in a qRT-PCR detection system (Thermo Fisher Scientific).

The threshold cycle (*C*t) values were normalized using a *C*t value derived from the following: ΔCt = Ct_target_ − Ct_GAPDH_; the expression of each RNA in the d-MSC group relative to that in the MSC group (fold change) was described using the following: 2^−ΔΔ*C*t^, where ΔΔ*C*t = Δ*C*t_d-MSC_ − Δ*C*t_MSC_.

### 4.3. In Vivo Study

#### 4.3.1. Manufacturing Artificial Trachea Using a 3D Bioprinter

The artificial trachea was cylindrical in shape and had five layers, an inner and outer diameter of 5 and 10 mm, respectively and a length of 15 mm. Polycaprolactone (PCL; MW = 45,000) (Sigma-Aldrich) was used as the supporting layer in the first, third and fifth layers and sodium alginate (Sigma-Aldrich) was used as the middle viscosity layer in the second and fourth layers from the inside. The PCL in the first and fifth layers were fabricated in diagonal grid patterns with micropores for ease of exchange of growth factors between cells and adjacent tissues, while the PCL in the third layer was fabricated in a helical form without pores to separate different cells in the second and fourth viscosity layers. PCL was placed in a 3D bioprinter (KIMM & Protek Korea, Daejeon, Korea) and dispensed through a 300 µm nozzle at a temperature of around 100 °C and a pneumatic pressure of 400 kPa. Moreover, the middle viscosity layer was fabricated in a helical form (epithelial cells in the second layer and MSC or d-MSC in the fourth layer). 1% calcium chloride solution was added to achieve the appropriate viscosity of sodium alginate hydrogel. Each hydrogel contained 1 × 10^7^ cells/10 mL. The cells encapsulated with alginate hydrogel was loaded into a disposable syringe of the same 3D bioprinter and dispensed through a 400 µm nozzle with a pneumatic pressure of 100 kPa at room temperature. The printing speed of all layer were 200 mm/min. The resulting artificial trachea was soaked in 5% calcium chloride solution for 30 min for gelation of bioprinted sodium alginate hydrogel and was subsequently transferred to a medium for cell preservation. All artificial tracheas were manufactured a day prior to surgical transplantation.

#### 4.3.2. Cell Distribution in the 3D Bioprinted Artificial Trachea

Cell distribution in alginate hydrogel was identified using CellTracker™ Fluorescent Probes (Thermo Fisher Scientific) according to the manufacturer’s protocol. Briefly, the dye vial was thawed at room temperature before opening and the dye product was dissolved in DMSO to a final concentration of 10 mM. Serum-free medium was added to the dissolved dye vial and the solution was warmed to 37 °C. The prepared cells were harvested by centrifugation and aspiration of the supernatant. The cells were re-suspended in a pre-warmed solution (epithelial cells in the red-dye vial and bMSC in the green-dye vial) and incubated for 30 min at 37 °C in a 5% CO_2_ incubator. Thereafter, centrifugation and removal of the supernatant were performed again and the cells obtained were used in the 3D bioprinting as previously described. The manufactured artificial tracheas were observed under a light microscope with appropriate optical filters according to the color of the dye.

#### 4.3.3. Scanning Electron Microscope (SEM)

The manufactured 3D bioprinted artificial trachea was imaged using a field emission scanning electron microscope with a backscattered electron image detector and an environmental secondary electron detector (JEOL. Ltd., Tokyo, Japan).

#### 4.3.4. Surgical Procedures for Tracheal Replacement Using 3D Bioprinted Artificial Trachea and Post Experimental Observation

**Figure 8:**
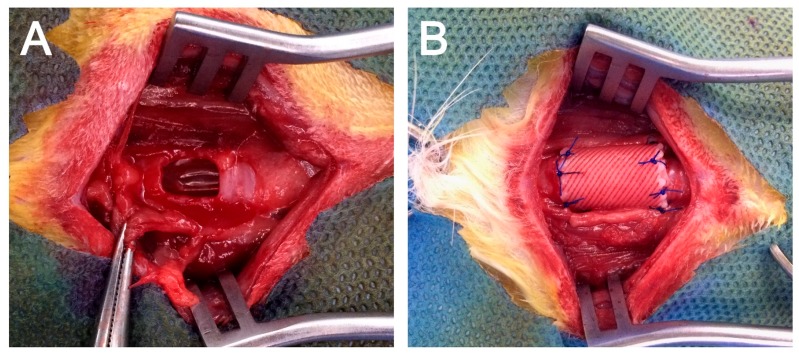
Application of 3D bioprinted artificial trachea. (**A**) Approximately 10 × 10-mm half-pipe-shaped tracheal defect on the ventral part of the trachea. (**B**) The defect was replaced with 3D bioprinted artificial trachea and sutured with 5–0 absorbable suture material.

After the administration of 5 mg/kg xylazine and 10 mg/kg Zoletil^®^ intramuscularly as pre-medications, 3.0 Fr tracheal tube intubation was performed and general anesthesia was maintained by isoflurane inhalation. The rabbits (*n* = 12) were placed in dorsal recumbent position with the neck slightly extended. The surgical region was shaved and disinfected. A vertical midline skin incision was made and the underlying trachea was carefully dissected from the cervical muscles. After cervical trachea exposure, a half-pipe-shaped partial tracheal resection (approximately 10 × 10 mm-size) was performed with a no. 15 scalpel. The defect was gently replaced with the pre-manufactured 3D bioprinted artificial trachea, which contains autologous epithelial cells and bMSC (*n* = 6, MSC group) or epithelial cells and chondrogenic-differentiated bMSC (*n* = 6, d-MSC group) (Figure 8). The artificial trachea was 15 mm in length (longitudinally sectioned and half-pipe-shaped to match the resected defect) and securely sutured with 5–0 Vicryl (Ethicon, Somerville, NJ, USA). Standard closure of the skin was performed and postoperative antibiotics were administered subcutaneously once a day for the first week. The animals were placed in cages at 20–24 °C and 40–60% humidity with a 12-h light/dark cycle and were fed standard laboratory rabbit food and water ad libitum. To evaluate the diameter of the airway, plain thoracic radiographs were obtained at after extubation, 4 and 12 weeks postoperatively with VXR-9M radiography system (DRGEM, Gwangmyeong-si, Korea). Bronchoscopic examinations with CV-260SL (Olympus, Tokyo, Japan) and computed tomography (CT) with Brivo 385 CT scanner system (GE Healthcare, Little Chalfont, UK) were performed at postoperative 12 weeks before sacrifice.

#### 4.3.5. Histopathology

All rabbits were euthanized at 12 weeks after transplantation. Tracheal segments including 5 mm of healthy tracheal tissue, both proximal and distal to the transplants, were harvested and fixed in 10% formalin. After fixation, the samples were embedded in paraffin blocks and cut (7-µm thick). All sections were obtained from the middlemost part of the samples including the transplants. The sections were deparaffinized, rehydrated and stained with hematoxylin and eosin (H&E) and safranin-O/fast green. Thereafter, the slides were observed under a light microscope. 

### 4.4. Statistical Analysis 

All data are expressed as mean ± standard deviation. Statistical analyses were performed using GraphPad Prism 5.0 software (GraphPad Software Inc., San Diego, CA, USA). Normal data distribution was determined using the Shapiro-Wilk test. A two-tailed Student’s unpaired *t*-test was used to compare the mean values of all study parameters. A *p* value < 0.05 was considered statistically significant.

## 5. Conclusions

In this study, we cultured and differentiated rabbit autologous bone marrow-derived mesenchymal stem cells into chondrocyte-like cells and confirmed its chondrogenic features by modified alcian blue absorbance test and qRT-PCR. The relative GAG accumulation level was increased as time goes by in chondrogenic differentiated mesenchymal stem cells. Also, chondrogenic-differentiation marker genes such as SOX-9, aggrecan, Col1α1 and Col2α1 expressions were upregulated in the d-MSC group compared with those in the MSC group. 

Moreover, we developed artificial trachea in a novel design by a 3d bioprinting technique, which contains two different kinds of cells (respiratory epithelial cells and chondrogenic-differentiated bone marrow-derived stem cells) in one scaffold. In our animal experiment on tracheal replacement, neo-cartilage formation was observed in a quite short period in d-MSC group, neo-epithelialization and neo-vascularization was identified as well. According to our study, differentiation of the bone-marrow derived mesenchymal stem cells considered to have more potential for tracheal cartilage regeneration.

## Figures and Tables

**Table 1:** Primer sequences used in qRT-PCR.

Gene	Primer Nucleotide Sequence
*ACAN*	Forward: 5-TCGAGGACAGCGAGGCC-3 Reverse: 3-AGAGATGTGCGATGTGGGAGCT-5
Col1α1	Forward: 5-GCGGTGGTTACGACTTTGGTT-3 Reverse: 3-AGTGAGGAGGGTCTCAATCTG-5
Col2α1	Forward : 5-GGCAATAGCAGGTTCACGTACA-3 Reverse: 3-TTCACCCCGTTCTGACAATAGC-5
*SOX9*	Forward: 5-CACACAGCTCACTCGACCTTG-3 Reverse: 3-GCTCTACTAGGATTTTATTGGCTT-5
*GAPDH*	Forward: 5-ATGGGGAAGGTGAAGGTCG-3 Reverse: 3-CCAGTGGTCCCGACGAAAAT-5
